# Pharmacological Chaperones and Protein Conformational Diseases: Approaches of Computational Structural Biology

**DOI:** 10.3390/ijms24065819

**Published:** 2023-03-18

**Authors:** Daniela Grasso, Silvia Galderisi, Annalisa Santucci, Andrea Bernini

**Affiliations:** Department of Biotechnology, Chemistry, and Pharmacy, University of Siena, 53100 Siena, Italy

**Keywords:** pharmacological chaperones, protein conformational diseases, computational structural biology, protein stability, transient pockets, pocket druggability, drug repurposing, virtual screening, molecular docking

## Abstract

Whenever a protein fails to fold into its native structure, a profound detrimental effect is likely to occur, and a disease is often developed. Protein conformational disorders arise when proteins adopt abnormal conformations due to a pathological gene variant that turns into gain/loss of function or improper localization/degradation. Pharmacological chaperones are small molecules restoring the correct folding of a protein suitable for treating conformational diseases. Small molecules like these bind poorly folded proteins similarly to physiological chaperones, bridging non-covalent interactions (hydrogen bonds, electrostatic interactions, and van der Waals contacts) loosened or lost due to mutations. Pharmacological chaperone development involves, among other things, structural biology investigation of the target protein and its misfolding and refolding. Such research can take advantage of computational methods at many stages. Here, we present an up-to-date review of the computational structural biology tools and approaches regarding protein stability evaluation, binding pocket discovery and druggability, drug repurposing, and virtual ligand screening. The tools are presented as organized in an ideal workflow oriented at pharmacological chaperones’ rational design, also with the treatment of rare diseases in mind.

## 1. Protein Conformational Diseases

Several biological mechanisms for the function of our organism rely on proteins, and a protein’s biological function is determined by its three-dimensional structure. Therefore, these molecules must be folded into a native state to function correctly. Still, the process of achieving native conformation is complex, which makes identifying the mechanism responsible for protein assembly central to structural biochemistry investigation. Indeed, an error in such a process could result in a misfolded protein or a missed quaternary assembly, leading to pathological effects. Known to cause cellular malfunctioning or death, altered proteins lose their function and lead to deleterious forms or aggregates [1,2]. Many factors contribute to the loss of the proper three-dimensional structure. Still, the amino acid composition remains the primary factor since mutations may destabilize the correct fold of a protein or stabilize a misfolded state [3,4,5].

Among protein-altering mutations, missense mutations are the most common [6]. A missense mutation occurs when one nucleotide base is substituted for another in a DNA sequence, resulting in a different codon and amino acid. The prevalence of missense mutations in any given gene is estimated at 2% [7]. Recent studies have focused on protein misfolding caused by missense mutations, which has led to the association with several serious diseases [4].

When proteins cannot fold into a native structure, a profound detrimental effect is expected, often resulting in disease. Known as protein conformational diseases, such disorders arise when proteins adopt abnormal conformations. Many people suffer from conformational diseases, significantly challenging human health [8]. The present review focuses on examples of rare types of conformational diseases, such as (with involved protein in parenthesis): Phenylketonuria (phenylalanine hydroxylase, PAH) [9,10], Alkaptonuria (1,2 homogentisate dioxygenase, HGD) [11,12], Transthyretin-related hereditary amyloidosis (transthyretin, TTR) [13], and the group of lysosomal storage diseases [14] including GM1-gangliosidosis (β-galactosidase), Tay-Sachs disease (β-hexosaminidase A), Sandhoff disease (β-hexosaminidases A and B), AB variant of GM2-gangliosidosis (GM2 activator protein), Fabry disease (α-galactosidase A), Gaucher disease (β-glucocerebrosidase), Pompe disease (α-glucosidase), mucopolysaccharidosis IIIC (heparan-α-glucosaminide N-acetyltransferase), and Batten disease (battenin). 

Molecular mechanisms that underlie many protein conformational diseases are very complex and multifactorial. Several phenomena are associated with conformational diseases, including loss of function, toxic gain of function, improper degradation, and improper localization. A loss-of-function mutation results in an impairment of the biological function, either complete (amorphic), which is analogous to protein null mutations, or partial (hypomorphic). Often, loss-of-function results from premature stop codons due to nonsense or frameshift mutations. The deleterious mRNA obtained usually produces no protein rather than a truncated polypeptide [15]. Hence, such mutations are usually of poor interest to the structural biologist, who is keener on missense mutations that change the amino acid sequence and result in protein products, although misfolded or non-functional. In structural terms, such mutations lead to (local) misfolding, active site disruption, or quaternary structure disruption. On the opposite side, the strengthening of protein–protein interactions induced by amino acidic mutation is a gain-of-function often resulting in a toxic effect. Mutations may increase protein activity (hypermorphic) or introduce a new function (neomorphic). Improper degradation relates to cellular degradation systems, such as autophagy; despite being essential for preventing the accumulation of misfolded proteins, it can sometimes lead to disease by over-actively degrading proteins that, despite their mutations, retain some functionality. The mislocalization of proteins arises from mutation destabilizing the correct fold and preventing the trafficking to the native subcellular compartment [16].

From a structural biology point of view, conformational diseases arise from a protein impairment to reach its native three-dimensional structure or stability. In turn, restoring the correct protein structure would rescue the biological activity and mitigate the disease; if molecules can mediate such a process, therapy development can be envisaged, but can protein rescue be mediated by molecular species? It happens in physiological conditions, where the acquisition of native conformation is made possible by specialized macromolecules called chaperones, which help proteins find their native functional conformation.

The chaperones to which we usually refer are proteins that assist the folding of other macromolecular structures and prevent the unwanted associations of unfolded polypeptide chains, both in physiological conditions and stress conditions. Since the tendency of a protein to aggregate increases in stress conditions, some chaperones are heat shock proteins (HSP), expressed in response to high temperatures or cellular stress. Physiological chaperons are essential in most cellular compartments, especially where proteins are subjected to potential stress, for example, mitochondrion, because of the low pH and the presence of degradation enzymes.

Based on such mechanisms, a new class of drugs is being exploited for protein rescue in conformational diseases: the pharmacological chaperones (PC). 

## 2. Pharmacological Chaperones

**Figure 1:**
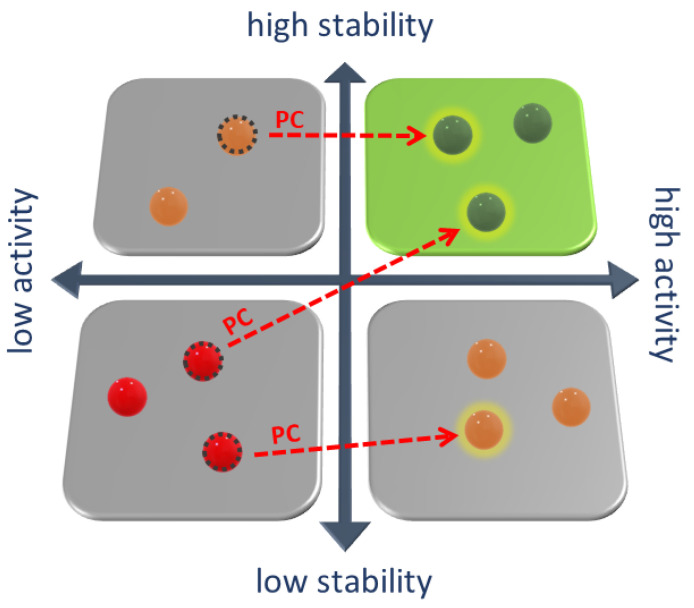
Native state protein has high activity and stability (green sphere). Misfolded proteins have lower stability, activity (orange spheres), or both (red). Pharmacological chaperones, binding with misfolded protein, increase its stability and activity. After binding with chaperones, proteins with high residual activity (**upper left square**) assume stability and activity similar to the wild-type. Proteins with lower residual activity (**lower left square**) assume high stability after binding with pharmacological chaperones, increasing the availability of protein in the cell.

Pharmacological chaperones (PC) are small molecule drugs designed to facilitate the correct folding of a protein and to re-establish its functionality [17,18] (see Figure 1). 

Similarly to physiological chaperones, these small molecules bind to poorly folded proteins, bridging non-covalent interactions (hydrogen bonds, electrostatic, and van der Waals) that were lost or loosened due to, e.g., missense mutations, thus strengthening the protein structure [19]. In addition, the low molecular weight of the PC gives the same advantages as other classes of drugs, such as molecular trafficking through membranes, reaching a variety of tissues [20], and oral availability [21].

Pharmacological chaperones should not be confused with chemical chaperones, such as trehalose and glycerol. The chemical chaperones are non-specific molecules; therefore, they bind to and stabilize all proteins. The unspecific binding and the required high concentration levels cause them to be unsuitable for therapeutic use.

Based on their molecular interaction with their target protein, PCs are classified into different types, namely, competitive inhibitors, enzyme cofactors, allosteric ligands, and alternative binders, described as follows. 

### 2.1. Competitive Inhibitors

Many of the PCs described so far have been competitive inhibitors of lysosomal storage disorders (LSDs) [22]. They create noncovalent hydrogen-bonding networks and van der Waals interactions in the same pocket as the enzyme substrate, stabilizing the protein structure. Consequently, when treating patients, a balance must be found between folding enhancement and enzyme inhibition by using subinhibitory concentrations. Moreover, PCs show a pH-dependent affinity to their target protein. The PC binds reversibly to its target protein in the endoplasmic reticulum (ER) at neutral pH, stabilizing and facilitating its transportation. An acidic pH in the lysosome allows dissociation between PC and its target protein, enabling metabolization. Dissociation at acidic pH is, therefore, crucial to broaden the narrow therapeutic index between enzymatic recovery and inhibition. Such behavior has been exploited to develop a PC for Fabry disease. Fabry disease is caused by variants in the GLA gene, coding for α-galactosidase A. Such enzyme, active in lysosomes, breaks down the fatty substance globotriaosylceramide, a glycosphingolipid. As a result of loss-of-function variants in the GLA gene, globotriaosylceramide builds up in cells throughout the body, particularly cells lining blood vessels in the skin and cells in the kidneys, heart, and nervous system. Fabry disease is characterized by the progressive accumulation of this substance in cells that results in various signs and symptoms. A low molecular weight iminosugar analog of the terminal galactose residue of GL-3, called migalastat, binds selectively and reversibly to the active site of various variants of α-galactosidase A enzyme [23,24,25]. Through this binding mechanism, and at levels below the inhibitory concentration, migalastat acts as a pharmacological chaperone, stabilizing variants of α-galactosidase A in the ER and facilitating proper trafficking to lysosomes [26]. Once in lysosomes, migalastat dissociates from α-galactosidase A due to the lower pH, allowing the enzyme to break down GL-3 [24,25,26]. Upon dissociation from the enzyme, migalastat is rapidly excreted from the body. Another example of a competitive inhibitor is iminosugar isofagomine, targeting and stabilizing mutants of β-glucosidase [27] and acting as PC in Gaucher disease [28].

### 2.2. Enzyme Cofactors

Enzyme cofactors are another type of PC. It may be beneficial for stabilizing misfolded proteins to increase the amount of the natural cofactor of an enzyme. The most common example of IEM is tetrahydrobiopterin (BH4), the cofactor for phenylalanine hydroxylase, which is defective in patients with phenylketonuria (PKU) [29,30,31]. About half of PKU patients respond well to this treatment [18], and BH4 has received FDA approval and is marketed under the commercial name Kuvan (see Table 1).

### 2.3. Allosteric Ligands

**Figure 2:**
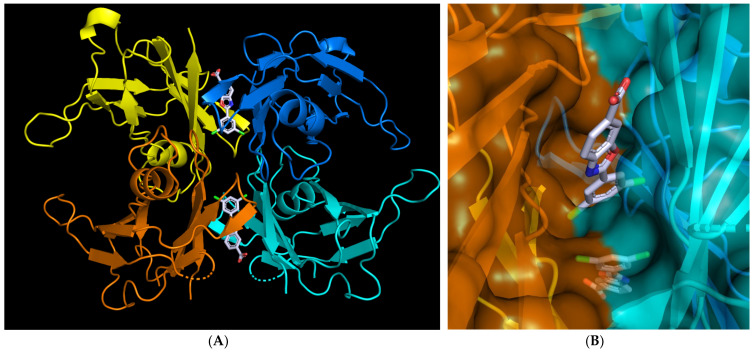
Three-dimensional structure of tafamidis-bound transthyretin (TTR). (**A**) The TTR homotetramer, arranged as a dimer of dimers (colored in yellow/orange and cyan/blue), is represented as ribbons; subunits assemble around a central channel that accommodates two drug molecules at the weak interacting surface of the dimers. (**B**) Close view of a tafamidis molecule filling the gap between surfaces of two dimers. Representations are made with PyMol using a structure from Protein Data Bank (PDB ID 3TCT, accessed on 10 January 2023).

PCs can also take the form of allosteric ligands. These PCs stabilize their targeted proteins by interacting in pockets other than the active site without impairing the function of the proteins, a clear advantage over competitive inhibitor PCs. A renowned example is the allosteric ligand Tafamidis (see [32,33,34,35] and Figure 2), already approved for transthyretin-related hereditary amyloidosis (ATTR) [36] treatment. 

ATTR is an autosomal neurodegenerative disease characterized by neuropathy and cardiomyopathy caused by variants in the transthyretin (TTR) gene. TTR carries vitamin A (retinol) and the hormone thyroxine (T4) throughout the body. It has a homotetrameric structure arranged as a dimer of dimers. In the presence of conformational variants, however, the equilibrium is tipped toward misfolded monomers, which are the origins of pathogenic aggregates and cell death. Patients carrying the most common variants of TTR, p.Val30Met, accounting for 85% of cases worldwide, were demonstrated to be protected by Tafamidis [37]. A successive study demonstrated that Tafamidis is also effective on eight variants [38] other than p.Val30Met: p.Asp38Ala, p.Gly47Ala, p.Leu58His, p.Thr60Ala, p.Phe64Leu, p.Ser77Phe, p.Ser77Tyr, and p.Ile107Val. The PC selectively binds to the two largely unoccupied thyroxine-binding sites of the tetramer located at the weak dimer–dimer interface (Figure 2), incrementing the number of interactions between protomers. Thus, in tafamidis-bound TTR, the weaker dimer–dimer interface is stabilized against dissociation, which is the rate-limiting step in forming amyloids [35]. Consequently, Tafamidis slows the progression of the disease. Many other conformational variants of the TTR protein have been treated with Tafamidis [39,40,41,42]. Tafamidis received approval from FDA and is marketed as Vyndamax (see Table 1).

**Table 1:** Pharmacological chaperones as therapeutics.

Name of Disease	Pharmacological Chaperones	Clinical Status
Transthyretin-related hereditary amyloidosis	Vyndamax (Tafamidis)	Market approved
Phenylketonuria	Kuvan (tetrahydrobiopterin or BH4)	Market approved
Fabry disease	Migalistat (1-deoxygalactonojirimycin or Galafold)	Market approved
Gaucher disease, Type 1	Afegostat tartrate (Isofagomine or AT2101)	Phase 2 NCT00446550
Gaucher disease, Type 1	Ambroxol	Phase 2 NCT03950050
Gaucher disease, Type 1	NCGC607	Preclinical cell-based study [43]
Pompe disease	Duvoglustat	Phase 2 NCT00688597
Pompe disease (late-onset)	Miglustat (AT2221) (with alglucosidase alfa, ATB200)	Phase 3 NCT03729362
Gangliosidoses, GM1	N-octyl 4-epi-β-valienamine	Preclinical in vivo study [44]
Gangliosidoses, GM1	1,5-dideoxy-1,5-iminoribitol C-glycoside	Preclinical cell-based study [45]
Gangliosidoses, GM2 Sandhoff disease Tay-Sachs disease	Pyrimethamine	Phase ½ NCT01102686
Mucopolysaccharidosis IIIC	Glucosamine	Preclinical in vivo study [46]
Batten disease	CS38	Preclinical cell-based study [47]

### 2.4. Alternative Binders

Research is becoming increasingly focused on finding binding sites other than the active ones to avoid interfering with the same biological function subject of the rescue strategy. In recent years, computational methods have been used for discovering and examining new hotspots that are not necessarily associated with active spots. Modulating the protein–protein interface by pharmacological chaperones is also becoming increasingly important in drug discovery, especially for diseases related to quaternary protein instability [48]. Such points will be addressed in the “Pocket prediction tools” and “Exploiting transient pockets” sections. 

Several other diseases are treated with pharmacological chaperones, with molecules already marketed and others under clinical trials of various phases. Conformational diseases and the PC targeted against are reported in Table 1. As for drug development in medicinal chemistry, a broad-spectrum screening of chemical libraries (high throughput screening, HTS) [49] is the faster and more efficient way to identify new-generation chaperones. However, PC development is often targeted at rare and neglected diseases, for which the development of new drugs is too long and expensive, with a risk of failure. For this reason, in the last years, the evolution of the drug repositioning approach has been proposed as a more suitable and less expensive way to identify lead compounds in rare and neglected disease drug development.4

## 3. Drug Repositioning

Drug repositioning (DR), also known as drug repurposing or retasking, is finding new indications for known drugs outside the scope of the original use [50]. The origin of drug repositioning derives from the capacity of some compounds to perturb multiple biological targets, allowing some drugs to be exploited for more than one disease [51,52]. 

DR usually focuses on molecules that have cleared phase I safety trials but have yet to show efficacy for the intended indication. Therefore, DR can probably skip the preclinical and phase I study [53], which can reduce the cost throughout drug development. The cost of repurposed drugs is typically reduced (50–60%), and their approval generally is quicker (3–5 years) [54,55]. Moreover, repurposed drugs achieve market approval at a higher rate than new drugs, giving companies an incentive to repurpose existing assets [54,56]. Nowadays, repositioning previously approved drugs is a common practice because of the increasing number of computational methods that reduce the number of potential compounds to be screened with high precision and sensitivity [51]. Therefore, this new method offers more significant advantages than de novo drug discovery, which is costly and time-consuming, sometimes with a scarce chance of success. 

Drug repositioning finds a convenient use in silico. Computational methods provide a valuable way for potential drug candidates to be identified and their interactions with proteins to be predicted. Such a method is called molecular structured-based virtual screening. It is commonly used in drug discovery by testing a large number of compounds and calculating the affinity with a target site. Further, it is known that many molecules have an affinity with more than one protein; thus, by screening known drugs, molecules active on targets of other diseases can possibly be found [51]. From a DR perspective, such virtual screening can be exploited for the out-licensing of drugs active on targets other than the original. The union of virtual screening and drug repositioning has been a significant step forward in drug discovery, especially for rare diseases [57]. 

The molecules that could be used for drug repositioning are [52]: (i) drugs in clinical trials that have a common mechanism of action with another disease; therefore, clinical trials for the new indication could be conducted in parallel with those for the original indication; (ii) drugs failed in a particular indication in clinical development without safety problems; (iii) drugs taken off the market for commercial reasons; (iv) drugs with patent near to expire; and (v) discovered drugs not launched in markets of developed areas. 

Ambroxol, a well-known molecule marketed as expectorant, is a case study of a drug repositioned as PC. In 2009 Maegawa et al. [58] carried out a library screening of FDA-approved drugs in search of stabilizers of variants of glucocerebrosidase (GCase), the deficient enzyme at the base of Gaucher disease (GD), the most prevalent lysosomal storage disease. Ambroxol was found to be a pH-dependent, mixed-type inhibitor of GCase and a stabilizer of the p.Asn370Ser and p.Phe213Ile variants. A few years later, the drug was proven effective against Gaucher variants [59]. Ambroxol improved or arrested the progression of neurological symptoms, such as dystonia and gait disturbances, in a clinical trial of Gaucher disease type 3 patients [60]. Ambroxol has been tested in Phase 2 clinical trial (see Table 1), and a recent observational study [61] has encouraged its off-label usage.

In summary, drug repositioning aims to extend a drug’s patent life and administer it to more than one pathology, increasing revenue. Indeed, drug repositioning contributes to improving pharma companies’ attention toward rare or neglected diseases. The FDA’s Office of Orphan Products Development aims to create strategies for treating and diagnosing rare diseases; also, it maintains the Rare Disease Repurposing Database [62].

## 4. Predicting the Pathogenicity of Variants and the Effect on Protein Stability

The first step in a bioinformatic workflow for developing a PC for protein rescue in a conformational disease framework is assessing the misfolding resulting from pathological variants and their extent. Many tools are available to accomplish this task, usually web tools (see Table 2) suitable for beginners. They should not be used as black-box anyway, and the results should be checked against experimental evidence and from more advanced simulation tools like molecular dynamics simulation (as illustrated later); also, comparing tool results using different approaches is valuable (see “Method” column in Table 2). For example, SIFT [63] is a multistep algorithm that uses sequence conservation and amino acid properties to predict whether an amino acid substitution is deleterious or neutral. Panther [64] is a valuable software system for predicting genes’ functions based on their evolutionary relationships. SNAP2 [65] is based on a neural network machine-learning algorithm, which distinguishes between neutral and non-neutral SNPs by taking evolutionary information derived from an automatically generated multiple sequence alignment. SNPs&GO [66] is a method that uses a protein sequence to predict whether single-point mutations can lead to diseases in humans. PolyPhen-2 [67] is a trained Naïve Bayes classifier that uses eight sequence-based and three structure-based predictive features automatically selected by its algorithm. FatHMM [68] predicts the functional effects of protein missense mutations by combining sequence conservation within hidden Markov models. VarMod [69] uses structural modeling of proteins, their ligand binding sites, and protein–protein interface sites to analyze non-synonymous single nucleotide variants to identify those that may play a role in diseases. MutPred2 [70] is a machine-learning-based method that integrates genetic and molecular data to predict the pathogenicity of amino acid substitutions and their molecular mechanisms. SNPdryad [71] builds a multiple sequence alignment using only protein orthologs and employs a random forest classifier to derive a deleterious prediction score. ENTPRISE [72] predicts human disease-associated amino acid mutation using a boosted tree regression machine-learning approach from sequence entropy. MutationAssessor [73] predicts the functional impact of amino acid substitutions in proteins, which is assessed based on the evolutionary conservation of the affected amino acid in protein homologs. Mupro [74] uses support vector machine approach combined with a local window centered around the mutated residue as input. CUPSAT [75] uses amino acid atom potentials and torsion angle distribution to assess the amino acid environment of the mutation site and the effect on stability. INPS [76,77] is based on physicochemical descriptors extracted from the protein sequence or structure to evaluate the impact of non-synonymous SNP on protein stability. SuSPect [78] uses 77 sequence-, structure-, and systems biology-based features to train a support vector machine to discriminate between disease-causing and neutral variants. SDM [79] is a computational method that analyzes the variation of amino acid replacements occurring at a specific structural environment for predicting protein stability upon mutation.

Similarly, mCSM-PPI2 [80] uses an optimized graph-based signature approach to evaluate the molecular mechanism of the mutation by modeling the effects of variations on the inter-residue non-covalent interaction network. The main difference with SDM is the presence, within mCSM-PPI2, of an interface analysis. DUET [81] combines two complementary approaches (mCSM and SDM) in an optimized predictor using support vector machines (SVM). mCSM-Membrane [82] is a machine-learning approach that uses graph-based structural signatures to test predictive models and analyze the impacts of mutations on the stability of membrane proteins and their possible association with diseases. mCSM-AB [83] is a machine-learning approach based on graph-based structural signatures that predict the change in antibody–antigen affinity upon introducing a single mutation. Finally, DynaMut2 [84] combines graph-based signatures with normal mode dynamics to predict the impact of actions on protein dynamics and stability resulting from vibrational entropy changes.

The described prediction tools are routinely used for pathogenicity prediction or rationalization of clinical evidence, and examples of specific applications to the field of PC are also reported. The web server for predictions of protein stability changes upon mutations (MUpro) has been employed to develop an index of responsiveness to pharmacological chaperones of variants of α-galactosidase in Fabry disease, scoring an 81% accuracy [85] for the PC Migalistat (now marketed as Galafold) vs. variants of α-galactosidase. Again for Fabry disease, PolyPhen-2 and SIFT have been used to support experimental data in the clinical evaluation of p.Asp313Tyr variant. The pathogenicity of such mutation has been debated since its first description in 1994 [86]. Recent studies converge to propose such a variant conducive to FD but with a milder phenotype and with later onset of symptoms [87,88,89,90]; predictions converge as well, reporting the variant as probably damaging (PolyPhen-2) or damaging (SIFT) [90]. 

## 5. Protein Instability Prediction by MD Simulation

The previously described methods are trained on datasets of protein sequences and structures and return scalar values, making them very effective in summarizing the effect of mutations and processing large amounts of proteins/mutations. However, biological activity relies not only on chemical factors but also on dynamics, regulating, e.g., active site adaptability, loop flexibility, domain swapping, and more, to investigate which time-dependent representation of wild-type/mutant proteins is required. Although experimental techniques may investigate protein dynamics (e.g., nuclear magnetic resonance relaxation), it is much more common and less expensive to adopt a computational biology approach to simulate molecular dynamics. In particular, molecular dynamics (MD) simulations based on the integration of Newtonian equations of motion, boosted by modern GPU platforms, can simulate the dynamics of a protein at an atomic resolution for a time range of up to milliseconds. Different timescale dynamics can be explored, from aromatic ring flipping to domain swap. The most used suites for MD simulations are GROMACS [91,92], Amber [93], NAMD [94], Desmond [95], and Tinker [96].

MD simulations start from a three-dimensional structure but in the case of ab initio folding. Experimental structures from the Protein Data Bank [97] are the ideal starting point, provided that the resolution is fair. Unlike the prediction methods already illustrated, prosthetic groups or post-translational modifications in MD simulation cannot be easily neglected. Their inclusion is a non-trivial task and must be evaluated. Similarly, missing ligands can be omitted or added by, i.e., docking (see “Virtual screening” section). MD simulations are used either to investigate local perturbation or overall folding instability introduced by mutations. The perturbation analysis is the most common: time evolution of the structure (the so-called trajectory) of the native protein and its mutants are compared either visually or by plot analysis of parameters like root mean square fluctuations, H-bond pattern, secondary structure conservation/disruption, etc. The comparison allows for identifying and quantifying structural deviances anywhere in the structure, including secondary structure, active sites, interfaces, etc. It must be noted that since the MD simulation of the mutant protein starts from a 3D structure, the simulation makes sense only if there is evidence that the mutant reaches a folded state, which is, as discussed before in the text, not straightforward, even for a missense mutation. Strong evidence is, of course, the presence of the mutant as PDB experimental structure, but also residual biological activity (e.g., enzymatic) can be evaluated, as well as the convergence of stability prediction by the methods mentioned above (see section “Predicting the pathogenicity of variants and the effect on protein stability”). When the mutant protein is unavailable as a PDB structure, the same can be modeled in silico starting from the native structure. Missense mutations are easily modeled with PyMol [98] molecular viewer. Its mutagenesis tool provides a point-and-click interface to substitute amino acids in the 3D view of the molecule. The mutated sidechain is modeled in situ according to a library of energetically favorite conformers; the conformer with the best score in terms of fewer clashes/favorable interactions is proposed; the user can also explore other solutions, e.g., by modifying the χ angles of sidechains.

Mutation-induced perturbation can be quantified more accurately by free energy calculation in alchemical transformation [99,100]. Here, the comparison of two MD trajectories (native/mutated) is replaced by a single trajectory where the interested amino acid sidechain structure is morphed into the new one along the MD run (hence the name alchemical). Energy evaluation of the resulting transformation trajectory with the Bennett acceptance ratio method (BAR) [101,102] or accelerated weighted histogram (AWH) [100,103] allows for free energy perturbation to be evaluated.

**Figure 3:**
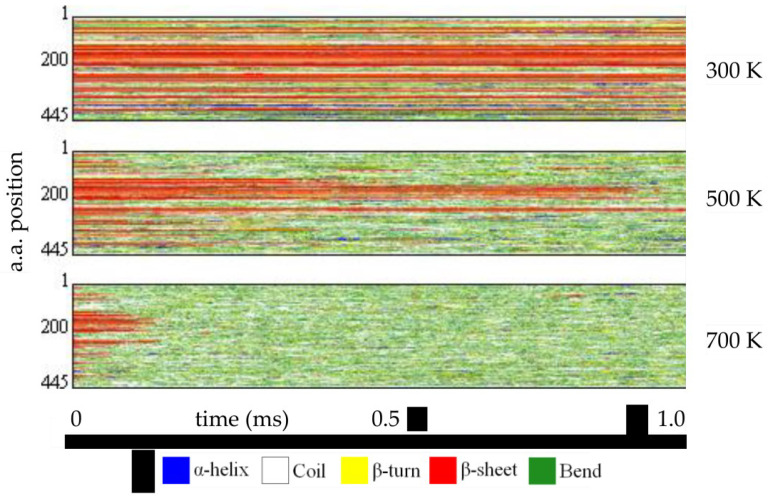
Example of unfolding MDs for wild-type HGD protomer from H. sapiens (Bernini A., unpublished data). The protein hydrated in a water box has been simulated at room temperature (300 K) and in increasing unfolding conditions (500 K and 700 K) for 1 ms with GROMACS and the amber force field. For each trajectory, the secondary structure content along the protein sequence (ordinate) has been evaluated as a function of time (abscissa) and plotted in different colors (see legend). A different rate of unfolding for the β-sheets is apparent by comparing the pictures.

When a mutation leads to misfolding, MD techniques may still help investigate the deleterious effects of the unfolding simulations. Indeed, experiments have demonstrated that unfolding MD simulations also shed light on folding, following the principle of microscopic reversibility, which states that folding and unfolding events are the same under the same conditions [104]. Thermal unfolding MD simulations obey the rules of Arrhenius. An increase in temperature does not modify the unfolding pathway but only its rate: the unfolding timescale is shortened as temperature increases. At the same time, the overall behavior and order of events are conserved [105]. In practice, the native and mutant proteins undergo multiple simulations at increasing temperatures, e.g., 300 K, 500 K, 700 K, and more, to achieve different unfolding rates and extents (see Figure 3).

The trajectories are then compared pairwise (native/mutant) with the analysis tools already described to highlight the structural moieties that unfold early/late. The influence of mutation can be evaluated. Although high-temperature unfolding MD simulation conditions appear unnatural, they benchmark well with NMR experimental data obtained at 298 K over the millisecond timescale [106]. 

Unfolding simulations have also performed well by replacing high temperatures with a denaturing environment like urea solution [107].

## 6. Pocket Prediction Tools

The topography of a protein structure is sculpted with many surface pockets and crevices, internal cavities, and channels. For example, ligands can bind to enzymes, and gas molecules can be routed through such microenvironments. Such microenvironments are best mapped by direct observation of a protein structure complexed with natural or engineered ligands (e.g., substrates, inhibitors), as found in PDB. Considering the reasons outlined in the introductory paragraphs, the trend over the past few years has been to identify PCs that bind to alternative sites of proteins rather than competing with the known ligand [20]. This can be experimentally achieved by, e.g., the use of molecular probes like in the multiple solvent crystal structure (MSCS) X-ray diffraction technique [108,109,110] or fragment-based NMR [111,112]. Alternative binding sites can also be discovered using bioinformatics tools mapping the surface/volume of a protein structure, allowing the search to be expanded to modeled proteins. This means virtually all the Uniprot (https://www.uniprot.org/ (accessed on 10 January 2023)) [113] entries missing 3D experimental structure, now provided with the AlphaFold [114] structure prediction (536,000 reviewed entries counted on 4 January 2022, limited to the Swiss-prot section and excluding those reporting experimental 3D structure). The tools for pocket/cavities discovery may rely on geometrical analysis, machine learning, or template-based approaches and are offered as web-based services (see Table 3). CASTp [115] is a metadatabase of topographies of PDB structures computed by grid-based geometry, but user PDB files can also be uploaded for computation. Results are displayed in a clean graphical viewer with pockets as bobble objects. 3DLigandSite [116] is a prediction tool using a template library of known binding sites; if the user cannot provide the 3D structure of the protein, the service retrieves it from AlphaFold [114] database or eventually models it with Phyre2 [117]. Prediction of protein-ligand binding residues is provided as a part of the intFOLD [118] modeling server, which uses the FunFOLD template-based algorithm; the online service starts from a protein sequence, and no custom PDB files are allowed. DeepSite [119] is a neural network-based predictor that works with PDB codes and user-supplied structures; results can be conveniently downloaded as comma-separated values (.csv) files and cavities as Gaussian format (.cube), suitable for more detailed analysis in, e.g., PyMol by representation as isomesh surface. COACH-D [120] is a ligand binding site predictor based on a support vector machine and refined by docking. The user can optionally upload a ligand structure that will be docked to the predicted pockets with Autodock Vina (discussed later), giving more targeted results. For modeling, proteins can be uploaded as PDB structure files or sequences in FASTA format.

Unlike the previous methods, Prankweb [121] (a web frontend to P2Rank) offers a template-free machine-learning method based on the prediction of surface ligandability, i.e., the convergence of favorable chemical interaction points on a solvent-accessible protein surface. 

The prediction of novel binding pockets on mutated proteins causing conformational diseases offers a valuable solution also when PC is already available but belongs to the competitive inhibitors class (see Section 2). The effectiveness of this kind of PC relies upon a delicate equilibrium with the substrate, which can be avoided by substituting the competitive PC with a stabilizer binding an alternative site, e.g., allosteric (see the Tafamidis/TTR example) or, even better, devoid of biological function. Thus, pocket prediction offers a solution for the development of PC of the second generation. For example, in Fabry disease research, such an approach allowed the discovery of a different druggable pocket located at the opposite side of the active site in α-galactosidase [122]. Such pocket is capable of binding 2,6-dithiopurine, and the binding stabilizes the enzyme in vitro and, noticeably, rescues the p.Ala230Thr variant that is not responsive to monotherapy with Migalistat (the competitive inhibitor PC for α-galactosidase, see Table 1). A similar effort has been carried out to overcome the drawbacks of inhibitory chaperones in Gaucher disease: a series of pyrrozolopyrazines [123] have been demonstrated to bind to a new pocket at a dimer interface and induce dimerization. 

## 7. Exploiting Transient Pockets for PC Binding

The methods illustrated in the previous paragraph allow for alternative pockets to be identified on a protein surface, but, despite the different approaches, they all share the limit of computing on a single structure, usually a conformation of minimum energy. As a result, the protein’s dynamics are neglected, a limit already highlighted for stability prediction methods. While molecular dynamics trajectories are usually explored focusing on binding sites and secondary structures, protein surfaces deserve more attention in the search for alternative binding sites, as they may “hide” transient pockets.

The term ”transient pocket” refers to a crevice opening at the protein surface due to side chains or backbone dynamics which is not detectable in the energy-minimized or averaged structure. When discussed in conjunction with drug binding, such pockets are also referred to as “cryptic” in literature [124]. Transient or cryptic pockets are valuable and provide a promising alternative to classical binding sites for drug development or repositioning.

The concept of protein transient pocket was first exploited as a target for disruptor of protein–protein interaction (PPI) since such interfaces are usually flat and strategies for drug design aiming at “classical” targets, e.g., G-protein-coupled receptors and enzymes, do not apply [125]. PPI investigations have shown that usually, in the center of the contact interface, a small subset of the residues complex contributes most of the free energy of binding. These hotspots have been exploited as receptor sites. Furthermore, once the PPI disruptor binds to the hotspot, the flat surface forms a groove, as has been observed in different protein–protein interaction systems, such as IL-2, Bcl-Xl, and HDM2 [125]. Moreover, molecular dynamics (MD) has shown that in the absence of PPI disruptors, proteins’ transient pockets open in less than one nanosecond [126].

The nature of the transient pockets has been studied with many alternative MD methods (NMA, CONCOORD, and tCONCOORD) [127] and different solvents (water and methanol) [128]. The result was that the number of transient pockets grew with methanol compared to those found in water, suggesting a lipophilic nature of the cavities. Moreover, it has been suggested that side chain movements are insufficient for a transient pocket to open; backbone dynamics are also necessary.

**Figure 4:**
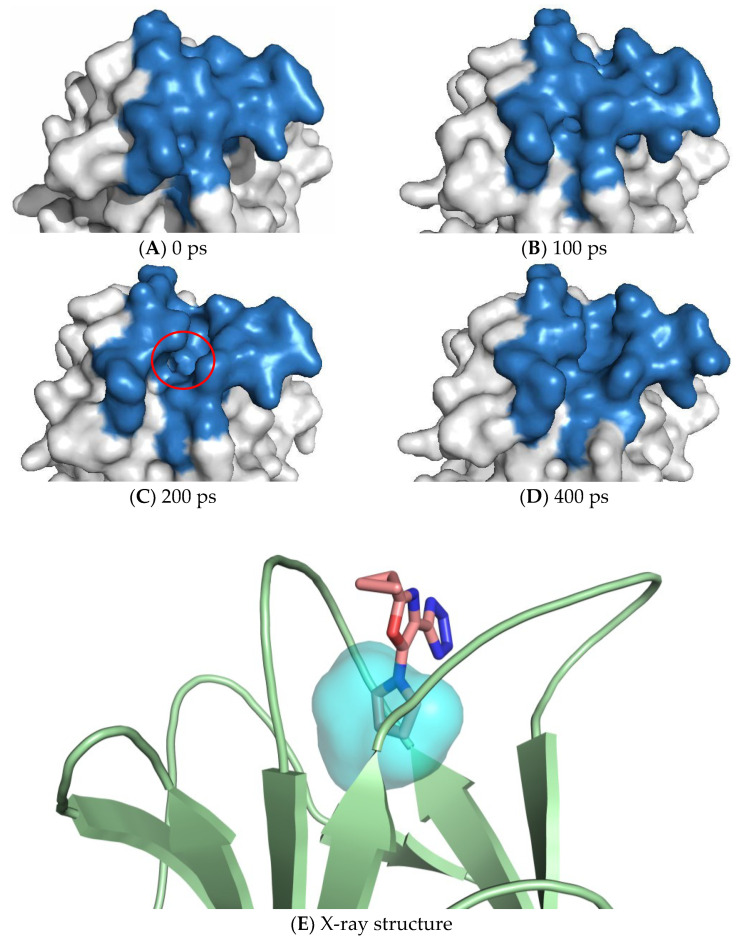
(**A**–**D**) Snapshots from an MD simulation showing the gradual opening/closing of a transient pocket on the surface region (blue) surrounding the Y220C mutation of the protein p53 (white). The red circle spots the transient pocket in the maximum depth conformation. (**E**) The transient pocket identified in the MD superposed to that occupied by the pyrrolic moiety of PhiKan7099 small molecule ligand (in pink) [132] in the experimental structure from PDB ID 5AOK (accessed on 10 January 2023).

In the last decade, transient pockets have been evaluated for the re-gain of activity of mutated proteins, as for the Y220C mutant of p53 tumor-suppression protein [129]. A first MD simulation and docking study identified a transient pocket opening transiently at Loop 1/β-strand 3 [130]. In successive research [129], several molecules were found binding at a transient pocket site. Such crevice has its maximum depth at the mutation site (Y220C) (Figure 4). A similar study has been carried out on the small chemokine CXCL12 (responsible for tumor progression and proliferation), where a transient pocket exploitable for binding small molecules has been observed both in MD simulation and in vitro by NMR experiment [131].

Furthermore, molecular dynamics simulation of the HIV-1 protease has shown a transient pocket with a lifetime longer than 1 μs that was used as the target for docking studies aiming to discover a new anti-AIDS drug [133].

The search for transient pockets available for small molecules is increasingly important indeed. For this reason, it requires effective programs to identify the latter. Programs for transient pocket prediction are usually MD trajectory-based, such as EPOS [126] and Mdpocket [134]. EPOS searches for transient pockets by analyzing a sequence of MD snapshots using the PASS pocket detection algorithm [135]. Similarly, MDpocket analyzes a set of structural snapshots sampled along the molecular dynamics trajectory by exploiting the fpocket program based on the Voronoi tessellation [134,136]. 

## 8. Druggability of Pockets

The approaches to pocket discovery described above open the way to a fundamental question: what causes a binding site to be a binding site? Indeed, in a drug-repositioning approach, finding alternative pockets is not enough; they must also be suitable for drug binding, i.e., “druggable”. Indeed, by looking at a binding pocket, the shape is the most evident trait, but the chemical properties cause it to be “binding”. The ability to efficiently bind a ligand is estimated from the possible number and types of non-covalent interactions that can be established, the so-called ligandability or druggability in medicinal chemistry research. A druggable pocket should reflect the typical features of a drug molecule, which are usually coded by Lipinski’s “Rule of Five” (Ro5) [137]. Ro5 describes the physicochemical characteristic required to be orally active for small molecules. According to the Ro5, a compound should possess the following properties to be active: (i) a molecular mass less than 500 Da; (ii) high lipophilicity: the octanol–water partition coefficient (LogP) should be less than 5; (iii) at most, five hydrogen bond donors; and iv) less than 10 hydrogen bond acceptors. All indicators are multiples of five indeed. A critical discussion on the effectiveness of the Ro5 covering two decades of research is reported in [138]. 

Predicting a protein pocket’s druggability is one of the most critical steps in drug discovery and development, now supported by many computational tools (Table 4). PockDrug [139] is a server relying on multiple pocket estimation methods allowing the prediction of pocket druggability of both apo and holo proteins with good accuracy. PockDrug combines four pocket estimation methods to evaluate the best pocket set from the ‘NonRedundant dataset of Druggable and Less Druggable binding sites’ (NRDLD) [140]. After model validation, PockDrug outputs druggability probability corresponding to the average of the seven best models and its standard deviation. Fpocket [136] is based on the geometric α-sphere theory; the Fpocket server consists of three software packages: (1) pocket identification using the concept of α-spheres (fpocket package); (2) pocket tracking along molecular dynamics trajectory(mdpocket package); and (3) collection of homologous structures using the pocket tracking (hpocket package). Through these three steps, the program allows the identification of both allosteric sites and transient pockets. DoGSiteScorer [141] is another server to analyze pockets’ geometric and physicochemical properties and predict their druggability; identifying possible pockets is performed using the protein’s heavy atom coordinates and calculating a density threshold. Based on a supporting vector machine (SVM), the algorithm proceeds for subpockets identification that are then blended into pockets. Protein–ligand interaction clusters (PLIC) [142] are a metadatabase that provides binding sites from the Protein Data Bank clustered by similarity using Markov clustering (MCL) [143] algorithm; by using computational tools, such as SPACE [144], fPocket [136], and Autodock [145], it also calculates various attributes of the binding sites, such as binding energy, shape, polarity, hydrophobicity, etc. CavityPlus [146] is a web server for pocket detection and functional analysis. The Cavity subroutine identifies cavities on protein surfaces using the NRDLD [140] dataset to train and validate the model. For each of them, CavityPlus provides pharmacophore modeling, allosteric site identification, and covalent ligandability prediction though the subroutines CavPharmer, CorrSite, and CovCys, respectively. PharmMapper Server [147,148] is a web server that, contrary to the others, starts from a small molecule probe and identifies potential targets using a pharmacophore mapping approach. PharmMapper relies on an in-house pharmacophore database obtained from the combination of other small molecule databases. In PharmMapper, target proteins with the highest fit scores between corresponding pharmacophore models and query compounds are reported as potential targets.

## 9. Virtual Screening of Compound Libraries in Search of PCs

Once a protein mutation and alternative binding site have been positively evaluated as a target for PC-mediated rescue, the hit discovery phase starts. Again, computational methods can be exploited to reduce time and cost in a workflow targeted to rare diseases; the list of compounds to be evaluated by experimental assay is slimmed down through simulated molecular docking of large compound databases [149]

A critical analysis of more than 400 studies involving virtual screening of small molecule libraries showed an overall hit rate ranging from 1% to 40% [150], comparable and somewhat superior to high throughput screening [151] both in terms of hit and screened compounds. A significant contribution to virtual screening comes from freely available databases of small molecule ligands (Table 5). Among the most popular, reporting drug-like, bioactive, and synthesizable compounds are ZINC [152], PubChem [153], DrugBank [154], ChEMBL [155], e-Drug3D [156], SuperDRUG2 [157], BindingDB [158], HMDB [159], and LIGAND [160]. The size of such molecular datasets, counting millions of compounds, has been expanded to billions of entries by enumerating synthetically feasible molecules (REAL: REadily Available for synthesis Library [161], 5.5B compounds at the time of writing) or by enumerating molecules in virtual chemical space (as in the Chemical Universe Database GDB-17 [162], reporting 166B compounds).

Molecular structures are usually available as 3D-coordinate file formats, such as sdf, mol2, and pdbqt (an extended format for pdb, including extra columns for atom charge and solvation energy). Other formats are becoming popular, such as the SMILES [163], which encodes the molecule topology into a lightweight ASCII string at the cost of losing conformation; on the contrary db2 format contains not one but multiple pre-calculated conformations [152] at the expense of larger file size. The protein molecule is the “target” of the screening and is usually provided in pdb format; the experimental or modeled structure or a snapshot from an MD simulation trajectory can be employed. Screening can be replicated over multiple trajectory snapshots to expand the target’s conformational sampling [164,165]. Usually, only the volume of the target containing the candidate pocket is screened, called the “docking box,” because of its simple shape. A box shape is used because of the easiness of mathematical representation in the pdb format, with three coordinates for the origin and three for the side lengths. 

Virtual screening can be performed with molecular docking simulation software that evaluates the ligand–target interaction energy according to a scoring function based on atom partial charge, desolvation energy, and others. The docking is flexible both on the ligand and target sides. Ligands are assigned rotatable bonds at system set-up, the same for protein but limited to amino acidic sidechains in the docking box. Among the freely available docking software, many belong to the Autodock family: Autodock 4 [166,167], Autodock Vina [168,169], AutoDockFR [170], FRED [171], EnzyDock [172], ICM [173], FlexX [174], Glide [175], GOLD [176], Mdock [177], and MOE [178]. Most of the mentioned software programs have also been optimized for parallel execution [179] and are readily available on high-performance computers with high scalability, allowing larger datasets to be screened.

The choice of docking software is connected mainly with the speed and accuracy of the scoring function, and performance comparison is largely covered in the literature [180,181,182,183]. Ease of usage is nowadays a standard, thanks to many graphical interfaces such as AutoDockTools [166], Raccoon v1.0 (AutoDock4) [184], and Raccoon v2.0 (AutoDock Vina) [184]. The typical output of a virtual screening with docking software is a list of compounds ranked by the score. The top-scorer is not necessarily the best hit [185]; it is then a good practice to take a top group of compounds and re-rank according to other scoring functions, molecular dynamics simulation-derived free energy of binding (e.g., MM/PBSA and MM/GBSA [186]), and, of course, experimental validation.

Molecular docking and MD simulation studies in the search for PC against conformational diseases have become more and more relevant in the recent literature. As an example, many can be found about the discovery of PC for β-glucocerebrosidase, the target protein in Gaucher disease [187,188,189,190,191]. Similar examples are reported for PC development rescuing β-hexosaminidase A (associated with Tay-Sachs disease) [192] and N-acetylgalactosamine-6-sulfate sulfatase (responsible for mucopolysaccharidosis IV A) [193].

Recently, machine learning [194,195,196,197] and deep learning by artificial intelligence [198,199,200] approaches have succeeded in molecular docking, enabling the virtual screening of libraries of up to billions of compounds. The first experimental validations of deep docking are also encouraging [201]. A breakthrough of AI in virtual screening similar to AlphaPhold [114] on structural biology is then envisaged and covered in Section 10.

## 10. The Impact of Artificial Intelligence

The re-emergence of artificial intelligence (AI) is having an impact on the drug-discovery process, e.g., by expanding the chemical space of virtual screening but also on de novo design of active compounds. Similar to drug-repositioning intent but with opposite approach, the advantage of such AI application in PC development for rare diseases is that they allow for scaffold hopping [202], which may help establish a competitive patent position [203], and are here described in their latest implementations in virtual docking and de novo design. 

Despite its benefits, virtual docking ultimately lags behind the rapid expansion of chemical databases which already exceed billions of records when going to virtual chemical space (e.g., the Chemical Universe Database GDB-17 [162] enumerates 166 billion organic small molecules). 

In order to address this challenge, AI is being introduced in the process and many platforms are already available. An example is the deep-learning platform Deep Docking (DD) [204]: small portions of ultra-large databases are used to train quantitative structure-activity relationship models that predict remaining entries’ scores, accelerating virtual screening 50 times. DD can now also take advantage of a convenient graphical user interface (DD-GUI) [205] for quick setups and outcome analyses of large-scale virtual screens. A similar approach of chemical space reduction, based on Bayesian optimization techniques, has been proposed to speed up the exploration of large molecular libraries by developing a surrogate model architecture [206]: in order to exclude the least promising compounds from evaluation, a surrogate structure–property relationship model can be created using the predicted affinities of a subset of the library. Inspired by natural processes, the ant colony optimization algorithm (ACO) is at the base of an artificial intelligence driven docking algorithm recently implemented in VirtualFlow [207], the VirtualFlow Ants [208]. The software showed almost linear scalability to 128,000 CPUs on a test library of a total of 1.44 billion compounds using KEAP1 protein as target. As an added value, it allows multiple re-scoring and on-the-fly conversion of structure formats. 

PyRMD [198] is a fully automated AI-powered ligand-based virtual screening tool that implements in Python the random matrix discriminant (RMD) method, capable of predicting whether a given small molecule could bind to a certain target. It requires only a set of active ligands on a specific target to train the algorithm, making it entirely ligand-based. A synthon-based approach, working in tight conjunction with REAL libraries [161], has been proposed in V-SYNTHES [209]. As the name suggests, the method is based on synthons; the synthons are those simple, charged chemical species that are formed during retrosynthetic analysis after imaginatively breaking down the target molecule.

While the goal of AI in the methods described above is to allow structure-based virtual screening to exploit compound libraries that are growing larger and larger, a different approach of “de novo” design is resurging thanks to machine intelligence development [210]. As the name implies, de novo design is the process of designing from scratch new chemical entities with desired (pharmacological) properties. Ideally, machine intelligence may jointly perform the three steps involved in the de novo design of molecules: generation, scoring, and optimization. 

In this view, the generative artificial intelligence approach [211] offers an AI model for the design of drug-like molecules. Such a deep-learning method is capable of “capturing” chemical determinants from a training set of drugs already known as active against a target protein and designing new chemical entities; it has been successfully applied to synthesizing novel active agonists of peroxisome proliferator-activated receptors. Similarly, generative adversarial networks have been proposed [212] for the de novo design of molecules from chEMBL [155] database. Reinforcement learning (RL) is at the base of ReLeaSE (Reinforcement Learning for Structural Evolution) strategy [213], targeted at generating chemical libraries of novel compounds optimized for either a single desired property or multiple properties. An up-to-date review of such a growing area of research can be found in [214].

## 11. Conclusions and Future Directions

**Figure 5:**
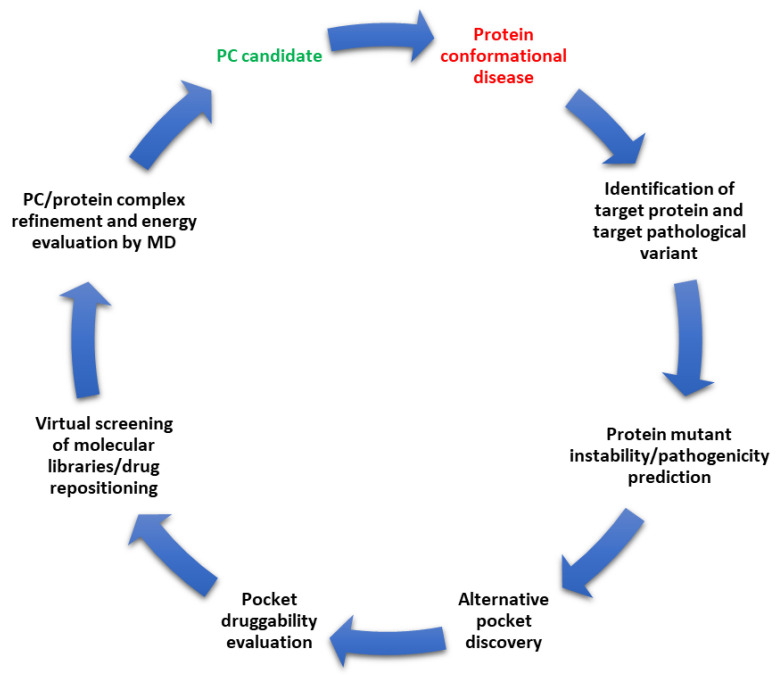
A general workflow for the application of computational structural biology to the development of pharmacological chaperones.

Conformational diseases result from misfolding of proteins, often due to missense pathological variants causing the loosening (or sometimes the strengthening) of the three-dimensional structure or quaternary assembly of the macromolecule. It has been shown that drug-like molecules may bind and stabilize the structure of poorly folded proteins, leading to the rescue of structure and activity, hence the name pharmacological chaperones. Furthermore, protein conformational diseases often superpose with rare genetic disorders, requiring strategies for lower drug development costs. The present review shows how the computational structural biology approach finds a convenient application in pharmacological chaperone development. We described a general workflow (Figure 5) that lines up several in silico tools for protein target discovery and pathogenicity prediction of variants (protein stability predictors and MD simulations) with those already used in rational drug design (pocket discovery, pocket druggability and virtual screening).

The next challenge for such a strategy will be replacing knowledge-based predictions with deep learning, which has already overperformed classical methods in structural biology.

## Figures and Tables

**Table 2:** Protein stability online prediction tools are listed along with the web address and method implemented.

Name	URL	Method
SIFT	https://sift.bii.a-star.edu.sg/ (accessed on 10 January 2023)	Sequence conservation and amino acids properties
Panther	http://pantherdb.org/tools/csnpScoreForm.jsp (accessed on 10 January 2023)	Evolutionary preservation
SNAP2	https://rostlab.org/services/snap/ (accessed on 10 January 2023)	Neural networks
SNPs&GO	https://snps-and-go.biocomp.unibo.it/snps-and-go/ (accessed on 10 January 2023)	Support vector machines
PolyPhen-2	http://genetics.bwh.harvard.edu/pph2/ (accessed on 10 January 2023)	Naïve Bayes classifier
FatHMM	http://fathmm.biocompute.org.uk/ (accessed on 10 January 2023)	Hidden Markov models
VarMod	http://www.wasslab.org/varmod/ (accessed on 10 January 2023)	Support vector machines
MutPred2	http://mutpred.mutdb.org/ (accessed on 10 January 2023)	Neural networks
SNPdryad	https://maayanlab.cloud/datasets2tools/landing/tool/SNPdryad (accessed on 10 January 2023)	Sequence alignment using protein orthologs
ENTPRISE	http://cssb2.biology.gatech.edu/ENTPRISE/ (accessed on 10 January 2023)	Sequence entropy and predicted protein structures
MutationAssessor	http://mutationassessor.org/r3/ (accessed on 10 January 2023)	Evolutionary preservation
MUpro	https://mupro.proteomics.ics.uci.edu/ (accessed on 10 January 2023)	Support vector machines
CUPSAT	http://cupsat.tu-bs.de/ (accessed on 10 January 2023)	Amino acid–atom potentials and torsion angle distribution
INPS	https://inpsmd.biocomp.unibo.it/inpsSuite (accessed on 10 January 2023)	Support vector machines
SuSPect	http://www.sbg.bio.ic.ac.uk/~suspect/ (accessed on 10 January 2023)	Support vector machines
SDM	http://marid.bioc.cam.ac.uk/sdm2/prediction (accessed on 10 January 2023)	Graph-based signatures
mCSM-ppi2	https://biosig.lab.uq.edu.au/mcsm_ppi2/ (accessed on 10 January 2023)	Graph-based signatures
DUET	http://biosig.unimelb.edu.au/duet/ (accessed on 10 January 2023)	Support vector machines
mCSM-Membrane	https://biosig.lab.uq.edu.au/mcsm_membrane/ (accessed on 10 January 2023)	Graph-based signatures
mCSM-AB	https://biosig.lab.uq.edu.au/mcsm_ab/ (accessed on 10 January 2023)	Graph-based signatures
DynaMut2	https://biosig.lab.uq.edu.au/dynamut2/ (accessed on 10 January 2023)	Graph-based signatures and normal mode dynamics

**Table 3:** Web tools for predicting protein pockets/cavities with the corresponding website and method.

Name	URL	Method
CASTp	http://sts.bioe.uic.edu/castp (accessed on 10 January 2023)	grid-based geometry
3DLigandSite	https://www.wass-michaelislab.org/3dligandsite (accessed on 10 January 2023)	template-based
IntFOLD	https://www.reading.ac.uk/bioinf/IntFOLD (accessed on 10 January 2023)	template-based
DeepSite	https://playmolecule.com/deepsite (accessed on 10 January 2023)	template-based, neural networks
COACH-D	https://yanglab.nankai.edu.cn/COACH-D (accessed on 10 January 2023)	consensus, SVM
PrankWeb	http://prankweb.cz (accessed on 10 January 2023)	template-free, random forest

**Table 4:** Freely available online tools for the prediction of protein pockets’ druggability and their web addresses. Methods applied are described in the text, along with references.

Name	URL
PockDrug	http://pockdrug.rpbs.univ-paris-diderot.fr/ (accessed on 10 January 2023)
Fpocket	https://fpocket.sourceforge.net/ (accessed on 10 January 2023)
DoGSiteScorer	https://proteins.plus/ (accessed on 10 January 2023)
CavityPlus	http://www.pkumdl.cn:8000/cavityplus/ (accessed on 10 January 2023)
PharmMapper	http://www.lilab-ecust.cn/pharmmapper/ (accessed on 10 January 2023)
PLIC	http://proline.biochem.iisc.ernet.in/PLIC/ (accessed on 10 January 2023)

**Table 5:** Free accessible databases of small molecule ligands and their corresponding web address.

Name	URL
ZINC20	https://zinc20.docking.org/ (accessed on 10 January 2023)
PubChem	https://pubchem.ncbi.nlm.nih.gov/ (accessed on 10 January 2023)
DrugBank	https://go.drugbank.com/ (accessed on 10 January 2023)
ChEMBL	https://www.ebi.ac.uk/chembl/ (accessed on 10 January 2023)
e-Drug3D	https://chemoinfo.ipmc.cnrs.fr/MOLDB/index.php (accessed on 10 January 2023)
SuperDRUG2	http://bioinf.charite.de/superdrug (accessed on 10 January 2023)
BindingDB	https://www.bindingdb.org/bind/index.jsp (accessed on 10 January 2023)
HMDB	https://hmdb.ca/ (accessed on 10 January 2023)
Ligand	https://www.genome.jp/kegg/compound/ (accessed on 10 January 2023)
REAL	https://enamine.net/compound-collections/real-compounds (accessed on 10 January 2023)
GDB17	https://gdb.unibe.ch/downloads/ (accessed on 10 January 2023)
